# QTL detection and candidate gene identification for prostrate growth habit in interspecific crosses of wild chrysanthemum (*Chrysanthemum yantaiense* × *C. indicum*)

**DOI:** 10.1093/hr/uhaf129

**Published:** 2025-05-21

**Authors:** Dawei Li, Yuxian Xu, Tongjun Zhou, Yuchao Tang, Hai Li, Ziyu Guo, Yilin Liang, Yuxin Wang, Yuyuan Chen, Ming Sun, Xuehao Fu

**Affiliations:** State Key Laboratory of Efficient Production of Forest Resources, Beijing Key Laboratory of Ornamental Plants Germplasm Innovation & Molecular Breeding, National Engineering Research Center for Floriculture, Beijing Laboratory of Urban and Rural Ecological Environment, Key Laboratory of Genetics and Breeding in Forest Trees and Ornamental Plants of Ministry of Education, School of Landscape Architecture, Beijing Forestry University, Beijing 100083, China; State Key Laboratory of Efficient Production of Forest Resources, Beijing Key Laboratory of Ornamental Plants Germplasm Innovation & Molecular Breeding, National Engineering Research Center for Floriculture, Beijing Laboratory of Urban and Rural Ecological Environment, Key Laboratory of Genetics and Breeding in Forest Trees and Ornamental Plants of Ministry of Education, School of Landscape Architecture, Beijing Forestry University, Beijing 100083, China; State Key Laboratory of Efficient Production of Forest Resources, Beijing Key Laboratory of Ornamental Plants Germplasm Innovation & Molecular Breeding, National Engineering Research Center for Floriculture, Beijing Laboratory of Urban and Rural Ecological Environment, Key Laboratory of Genetics and Breeding in Forest Trees and Ornamental Plants of Ministry of Education, School of Landscape Architecture, Beijing Forestry University, Beijing 100083, China; State Key Laboratory of Efficient Production of Forest Resources, Beijing Key Laboratory of Ornamental Plants Germplasm Innovation & Molecular Breeding, National Engineering Research Center for Floriculture, Beijing Laboratory of Urban and Rural Ecological Environment, Key Laboratory of Genetics and Breeding in Forest Trees and Ornamental Plants of Ministry of Education, School of Landscape Architecture, Beijing Forestry University, Beijing 100083, China; State Key Laboratory of Efficient Production of Forest Resources, Beijing Key Laboratory of Ornamental Plants Germplasm Innovation & Molecular Breeding, National Engineering Research Center for Floriculture, Beijing Laboratory of Urban and Rural Ecological Environment, Key Laboratory of Genetics and Breeding in Forest Trees and Ornamental Plants of Ministry of Education, School of Landscape Architecture, Beijing Forestry University, Beijing 100083, China; State Key Laboratory of Efficient Production of Forest Resources, Beijing Key Laboratory of Ornamental Plants Germplasm Innovation & Molecular Breeding, National Engineering Research Center for Floriculture, Beijing Laboratory of Urban and Rural Ecological Environment, Key Laboratory of Genetics and Breeding in Forest Trees and Ornamental Plants of Ministry of Education, School of Landscape Architecture, Beijing Forestry University, Beijing 100083, China; State Key Laboratory of Efficient Production of Forest Resources, Beijing Key Laboratory of Ornamental Plants Germplasm Innovation & Molecular Breeding, National Engineering Research Center for Floriculture, Beijing Laboratory of Urban and Rural Ecological Environment, Key Laboratory of Genetics and Breeding in Forest Trees and Ornamental Plants of Ministry of Education, School of Landscape Architecture, Beijing Forestry University, Beijing 100083, China; State Key Laboratory of Efficient Production of Forest Resources, Beijing Key Laboratory of Ornamental Plants Germplasm Innovation & Molecular Breeding, National Engineering Research Center for Floriculture, Beijing Laboratory of Urban and Rural Ecological Environment, Key Laboratory of Genetics and Breeding in Forest Trees and Ornamental Plants of Ministry of Education, School of Landscape Architecture, Beijing Forestry University, Beijing 100083, China; State Key Laboratory of Efficient Production of Forest Resources, Beijing Key Laboratory of Ornamental Plants Germplasm Innovation & Molecular Breeding, National Engineering Research Center for Floriculture, Beijing Laboratory of Urban and Rural Ecological Environment, Key Laboratory of Genetics and Breeding in Forest Trees and Ornamental Plants of Ministry of Education, School of Landscape Architecture, Beijing Forestry University, Beijing 100083, China; State Key Laboratory of Efficient Production of Forest Resources, Beijing Key Laboratory of Ornamental Plants Germplasm Innovation & Molecular Breeding, National Engineering Research Center for Floriculture, Beijing Laboratory of Urban and Rural Ecological Environment, Key Laboratory of Genetics and Breeding in Forest Trees and Ornamental Plants of Ministry of Education, School of Landscape Architecture, Beijing Forestry University, Beijing 100083, China; State Key Laboratory of Efficient Production of Forest Resources, Beijing Key Laboratory of Ornamental Plants Germplasm Innovation & Molecular Breeding, National Engineering Research Center for Floriculture, Beijing Laboratory of Urban and Rural Ecological Environment, Key Laboratory of Genetics and Breeding in Forest Trees and Ornamental Plants of Ministry of Education, School of Landscape Architecture, Beijing Forestry University, Beijing 100083, China

## Abstract

The prostrate growth habit is an important ornamental trait in ground-cover chrysanthemum, offering high aesthetic value, strong lodging resistance, and excellent landscape greening capability. However, the genetic basis underlying this trait in chrysanthemum remains largely unclear. In this study, we utilized the prostrate-type *Chrysanthemum yantaiense* (tetraploid), the erect-type *C. indicum* (tetraploid), and their 199 F_1_ hybrid progenies to construct a high-density genetic linkage map through genotyping-by-sequencing. The biparental linkage maps included 4614 and 5180 SNP markers, with an average marker distance of 0.84 and 0.73 cM, respectively. After four years of phenotypic evaluation and one year of dynamic trait measurement in progenies for traits related to prostrate growth habit, we confirmed a stable quantitative trait locus (QTL) located on LG1–1 among co-localized QTLs using KASP markers. This QTL explained up to 20.13% of the phenotypic variation. As a result, a total of 44 genes were identified as candidate due to their tightly linkage with the peak QTL marker, Tag16173. Further phytohormone measurement, gene expression analysis, and transgenic studies confirmed that one of these candidates, the D type cyclin-encoding gene *CyCYCD3;1*, played a key role in the formation of prostrate growth habit in *C. yantaiense*. Our results not only enhance the understanding of the molecular mechanisms behind prostrate growth habit but also provide valuable molecular markers for improving plant architecture-related traits in chrysanthemum breeding.

## Introduction

Plant architecture, encompassing key agronomic traits like plant height, branch angle, and growth habit, is a fundamental factor influencing both crop yield and ornamental value [1–3]. The prostrate growth habit, a distinct form of plant architecture, is characterized by low height, multiple tillers, and an outward-spreading growth pattern, allowing plants to extend over a larger area. The prostrate growth habit enhances lodging resistance, making plants more resilient to wind, adverse weather, and trampling, while also contributing to their superior landscape greening capability [4]. Investigating how prostrate growth habit was formed, therefore, is not only the key to understanding how plants adapt to extreme environments but also has theoretical and practical significance for breeding varieties with this desirable trait.

Our knowledge of prostrate growth habit primarily comes from studies of crops, such as rice (*Oryza sativa*), peanuts (*Arachis hypogaea*), and barley (*Hordeum vulgare*) [1, 4–6]. In wild rice (*O. rufipogon*), the prostrate growth habit is believed to be controlled by the *PROSTRATE GROWTH 1* (*PROG1*) gene; disruption of its function or inactivation of its expression resulted in erect growth [4]. Additionally, studies on the rice agravitropic mutant *lazy1* has shown that *LAZY1* acts as a negative regulator of polar auxin transport, with its dysregulation likely contributing to the tiller-spreading phenotype [1, 7]. In barley, the *sdw1*/*denso* has been recognized as a key determinant of semi-dwarfing traits, as it responds to gibberellic acid, and recessive alleles at the *denso* locus led to a prostrate growth habit [5]. In peanuts, the construction of a high-density genetic linkage map and QTL analyses identified 19 candidate genes associated with prostrate growth habit, most of which were involved in the cytokinin-activated signaling pathway [8]. Despite these advances, our knowledge about the genetic and physiological mechanisms underlying prostrate growth habit is far from being complete.

Chrysanthemum is a globally important ornamental plant, renowned for its aesthetic appeal as well as its uses in food, tea, and traditional medicine [9]. With rising living standards and a growing demand for low-carbon, resource-efficient landscaping solutions, there is an increasing consumer preference for novel, eco-friendly ornamental plants. As a result, the ground-cover chrysanthemum, known for their enhanced lodging resistance, superior landscape greening potential, and reduced landscaping costs compared to traditional cultivated chrysanthemum, are becoming an increasingly attractive option for modern garden designs. To achieve this aim, the beneficial characteristics of wild chrysanthemum species have been explored for integration into modern cultivars. One promising germplasm is *Chrysanthemum yantaiense*, a wild species found near the sea, which exhibits a superior prostrate growth habit, high stress resistance, and strong ground-covering ability [10]. While introgressing alleles from wild species into cultivated chrysanthemum is feasible, traditional hybrid breeding faces significant challenges due to the long generation time, gametophytic self-incompatibility, and high levels of heterozygosity in chrysanthemum [11].

An effective approach to addressing this challenge is to develop high-quality molecular markers for wild chrysanthemum germplasm and employ them in genetic breeding [12]. Recent genetic studies have focused on constructing high-density genetic maps and developing efficient molecular markers to facilitate marker-assisted selection (MAS) for target traits in chrysanthemum [13–17]. For example, Zhang *et al.* used 896 SRAP markers to construct a genetic linkage map for chrysanthemum, and detected 12 QTLs linked to inflorescence traits [13]. Similarly, Fan *et al.* developed a genetic map with 264 SSR markers, and identified 36 QTLs related to inflorescence and leaf traits [16]. With the advances in sequencing technology, high-precision SNP markers are increasingly replacing other molecular markers in construction of high-density genetic maps. For instance, van *et al.* (2017) genotyped an F_1_ population with an SNP array of 183 000 markers, constructing the first high-density genetic map for chrysanthemum and identifying QTLs associated with the flower color and flowering time [14]. Likewise, Song *et al.* employed high-throughput sequencing to create a high-density genetic map spanning 4301.5 cM, covering 6452 SNP markers, and identified three major QTLs for corolla tube merging and four major QTLs for the relative number of ray florets [17]. These studies all focus on hexaploid cultivated chrysanthemum, with an emphasis on floral traits, while the inheritance of prostrate growth habit remains largely unexplored.

**Figure 1:**
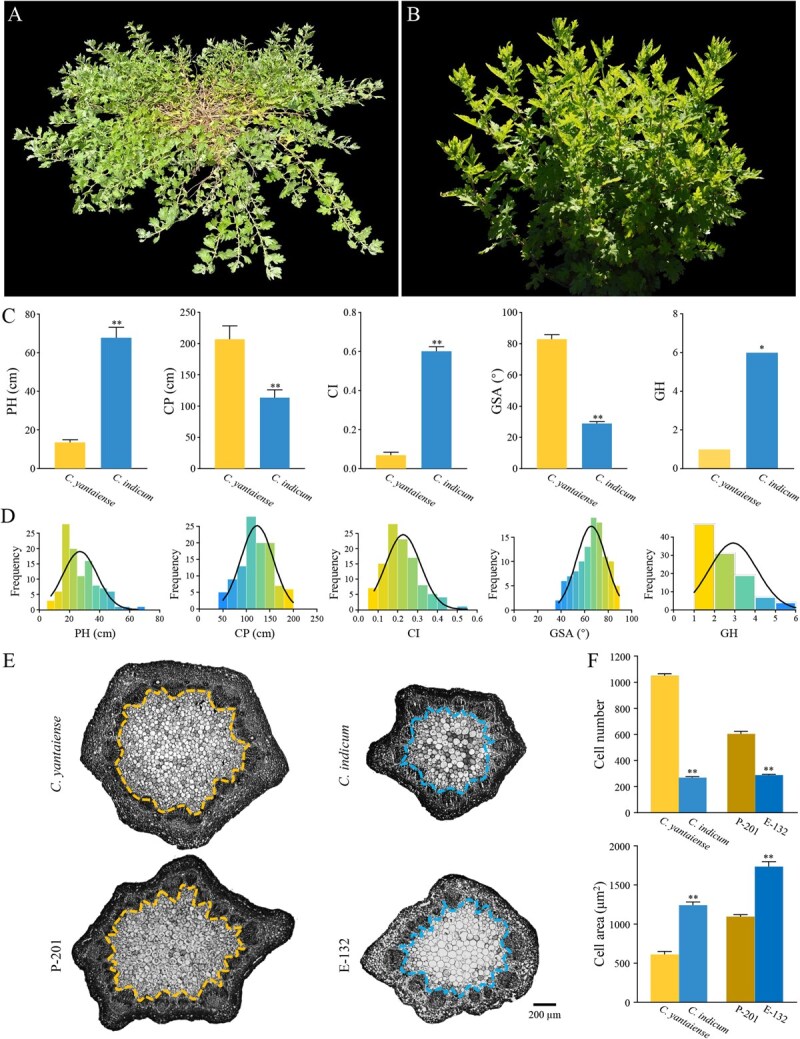
Phenotypic evaluation of prostrate growth habit related traits in the *Chrysanthemum yantaiense*, *C. indicum*, and the F_1_ mapping population. A *C. yantaiense*. B *C. indicum*. C Plant height (PH), crown width of the plant (CP), creeping index (CI), gravitropic set-point angle of branching (GSA), and growth habit (GH) of *C. yantaiense* and *C. indicum*. D Distribution of PH, CP, CI, GSA, and GH in the F_1_ mapping population (2020). E Transverse sections of apical stem of *C. yantaiense*, *C. indicum*, P-201 and E-132. Bar = 200 μm. F Cell number and cell area in the apical stem of *C. yantaiense*, *C. indicum*, P-201, and E-132. Data are presented as means ± standard error from three independent replications. Statistical significance was determined by Students *t*-test: ^**^P < 0.01.

In this study, we generated a hybrid population by crossing the prostrate-type *C. yantaiense* (tetraploid) with the erect-type *C. indicum* (tetraploid) (Fig. 1A and B) and used the 199 F_1_ hybrid progenies to construct a high-density genetic linkage map through genotyping-by-sequencing. By conducting extensive genetic, morphological, physiological, and functional studies, we attempted to: (i) identify QTLs associated with five traits related to prostrate growth habit, including plant height (PH), crown width of the plant (CP), creeping index (CI), gravitropic set-point angle of branching (GSA), and growth habit (GH); (ii) explore the physiological and genetic mechanisms underlying prostrate growth habit in *C. yantaiense*; and (iii) provide valuable molecular markers for improving plant architecture-related traits in future chrysanthemum breeding.

## Results

### Phenotypic features of *C. yantaiense*, *C. indicum*, and their progenies

To understand prostrate growth habit differences among the parents and progenies, five related traits, including plant height (PH), crown width of the plant (CP), creeping index (CI), gravitropic set-point angle of branching (GSA), and growth habit (GH), were evaluated in the F_1_ mapping population and the two parents over four years (Table 1). In the parents, *C. yantaiense* showed 5.01- and 8.57-fold lower PH and CI compared to *C. indicum*, while its CP and GSA were 1.82- and 2.87-fold higher (Fig. 1C). Additionally, based on our classification, *C. yantaiense* exhibited the highest GH level, whereas *C. indicum* had the lowest (Fig. 1C). In the progenies, we found that all measured traits showed continuous distributions, indicating distinct quantitative genetic characteristics (Figs 1D and S1). The mean of the coefficient of variation (CV) for PH and CI was 42.36% and 40.28%, while those for CP, GSA, and GH were 28.54%, 19.03%, and 38.85%, respectively (Table 1). By utilizing the SEA software for inheritance model analysis, the results suggested that PH was controlled by one or two pairs of additive-dominant major genes, CI was controlled by one pair of additive-dominant major genes, GSA was governed by two pairs of equal-dominant major genes, while CP and GH were controlled by polygenes and two pairs of major genes, respectively (Table S1). The mean heritability of major gene for traits PH, CP, CI, GSA, and GH was 67.19%, 76.25%, 49.98%, 73.44%, and 75.15%, respectively (Tables 1 and S1). Further correlation analyses suggested that, except for CP and GH, and CP in 2020, the five traits, as well as traits across different years, were significantly correlated (Fig. S2).

**Table 1:** Statistics of the five prostrate growth habit-related traits in the F_1_ mapping population over four years.

**Trait**	**Year**	***C. yantaiense***	***C. indicum***	**Significant difference**	**F**_**1**_ **mapping population**			
					**Mean**	**CV**	**Range**	**Heritability (Major-Gene) (%)**
PH(cm)	2019	14.20	73.50	Yes, *P* < 0.001	28.60	0.42	6.80–69.80	67.19
	2020	13.70	75.70		26.92	0.42	7.70–65.10	
	2021	16.40	52.40		29.79	0.43	9.20–62.90	
	2022	9.90	69.80		31.55	0.42	10.20–79.30	
CP(cm)	2019	205.00	121.20	Yes, *P* < 0.01	102.18	0.32	42.70–193.10	76.25
	2020	211.60	134.60		122.60	0.28	50.20–199.90	
	2021	154.30	79.20		100.98	0.25	40.50–164.40	
	2022	257.90	120.80		135.10	0.29	46.40–228.20	
CI	2019	0.07	0.61	Yes, *P* < 0.001	0.29	0.39	0.10–0.64	49.98
	2020	0.06	0.56		0.23	0.39	0.08–0.55	
	2021	0.11	0.66		0.31	0.46	0.10–0.94	
	2022	0.04	0.58		0.24	0.37	0.09–0.59	
GSA(°)	2020	81.73	27.30	Yes, *P* < 0.001	65.68	0.19	37.87–89.37	73.44
	2021	78.87	31.37		68.04	0.19	28.13–88.97	
	2022	88.33	28.17		59.19	0.19	29.30–81.77	
GH	2019	1.00	6.00	Yes, *P* < 0.05	2.32	0.51	1.00–6.00	75.15
	2020	1.00	6.00		2.93	0.40	1.00–6.00	
	2021	1.00	6.00		3.22	0.36	1.00–6.00	
	2022	1.00	6.00		3.00	0.28	1.00–6.00	

To further investigate the histological differences between prostrate and erect stems, the apical stems of two parents and two progenies with extreme phenotypes (i.e. P-201 and E-132) were examined. Our observations revealed that the stem pith of *C. yantaiense* and P-201 contained a significantly higher number of cells compared to those of *C. indicum* and E-132 (Fig. 1E and F). In contrast, *C. indicum* and E-132 exhibited significantly larger cell areas (Fig. 1E and F). These findings suggest that the difference in both cell number and cell area in the stem pith may contribute to the formation of prostrate growth habit.

### Construction of the high-density linkage map

**Figure 2:**
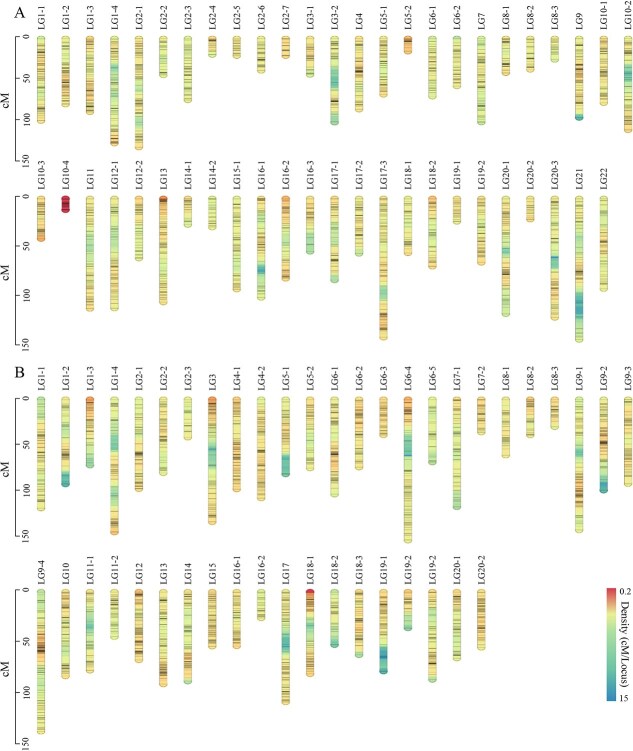
Bi-parental genetic linkage maps of the F_1_ mapping population. A The maternal genetic linkage map. B The paternal genetic linkage map. Markers are depicted as black bars.

To identify single nucleotide polymorphisms (SNPs) or insertion and deletion variations (INDELs) used for map construction, the two parents and 199 F_1_ progenies obtained in 2018 were genotyped using genotyping-by-sequencing (GBS). A total of 135 Gb raw data were generated, with average coverage depths of 45.79 × for *C. yantaiense*, 41.74 × for *C. indicum*, and 47.67 × for the progenies. Clean reads were further mapped to the GBS reference, yielding 33 794 molecular markers, including 31 500 SNPs and 2294 INDELs. After filtering out markers with segregation distortion and low quality, 9794 high-quality markers were retained and used to construct separate genetic maps of the two parents (Fig. 2; Table S2). The female parent, *C. yantaiense*, had a genetic map distance of 3884.9 cM, comprising 4614 molecular markers distributed across 22 linkage groups (LGs) (Fig. 2A; Table S3). The number of markers per linkage group varied from 71 to 575, with the largest gap being 14.5 cM in LG 21–1, and the average genetic distance between markers being 0.84 cM (Fig. 2A). For male parent *C. indicum*, 5180 molecular markers were identified across 20 LGs, spanning a distance of 3756.3 cM (Fig. 2B; Table S4). The number of molecular markers per group ranged from 131 in LG 15 to 648 in LG 9, with an average genetic distance between markers being 0.73 cM (Fig. 2B; Table S4). Taken together, these findings suggest we have obtained two high-density linkage maps, which are suitable for identifying QTLs associated with prostrate growth habit.

### Identification of QTLs associated with prostrate growth habit

To identify QTLs associated with prostrate growth habit, we performed QTL mapping on the F_1_ mapping population using the two high-density linkage maps and phenotypic data of five related traits collected over four years (2019–2022). A total of 111 QTLs for PH, CP, CI, GSA, GH, and dynamic traits were identified, and mainly distributed across LGs 1, 2, 3, 10, 11, 20 in the maternal genetic map and LGs 1, 9, 11, 13, 14, 18 in the paternal genetic map (Table S5). Notably, 53 of these were classified as dynamic QTLs, as they were identified by combining the genetic maps with phenotypic data of PH, CP, and CI across eight growth stages in 2021. Specifically, 37 QTLs associated with PH were identified on maternal LGs 1, 2, 6, 10, and 11, and on paternal LGs 1, 9, 11, 13, 14, 18, and 19, explaining up to 21.17% of the phenotypic variation (Table S5). For CI, 38 QTLs were found on maternal LGs 1, 2, 3, 10, 11, and 20 and on paternal LGs 1, 7, 9, 11, 13, and 20, accounting for 6.88% to 26.97% of the phenotypic variation (Table S5). In the case of GSA, 13 QTLs were detected on maternal LGs 1, 9,11, and 20 and on paternal LGs 9 and 11, explaining 8.41% to 23.03% of the phenotypic variation (Table S5). For GH, 14 QTLs were identified on maternal LGs 1, 2, 11, and 12, and on paternal LGs 2, 9, 11, and 18, contributing 7.24% to 21.10% of the phenotypic variation (Table S5). Furthermore, nine QTLs associated with CP were identified on maternal LGs 12, 13, and 22 on paternal LGs 1, 9, and 18, accounting for 7.92% to 12.39% of the phenotypic variation (Table S5).

**Figure 3:**
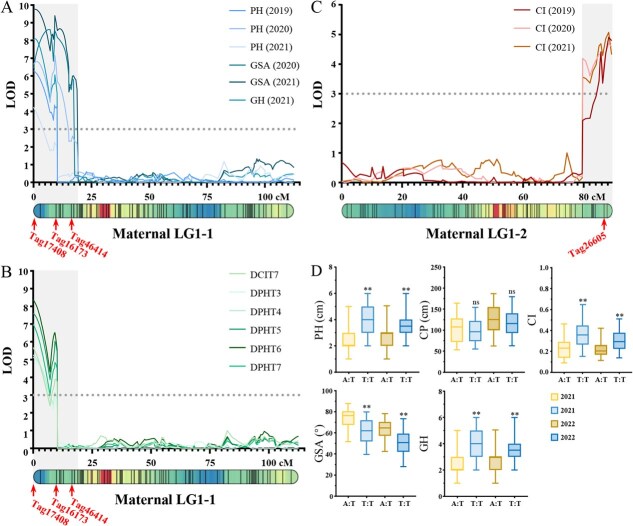
Co-localized QTLs of prostrate growth habit-related traits. A Co-localized QTLs and the peak markers on LG1–1. B Co-localized dynamic QTLs and the peak markers on LG1–1. C Co-localized QTLs and the peak marker on LG1–2. D PH, CP, CI, GSA, and GH of validation population under different genotypes (A:T and T:T) of KASP_16173. Statistical significance was determined by Students *t*-test: ^**^P < 0.01; ‘ns’ denotes nonsignificance.

Notably, among these QTLs, 43 were colocalized across thirteen regions on the genetic map, with four regions considered stable due to their identification over two years or across more than three traits at the same location (Fig. 3A–C). Interestingly, all four stable QTLs were located on maternal LG1 (Fig. 3A–C). The first stable QTL region was localized at the top of LG 1–1 (0–4.0 cM), encompassing 12 QTLs (i.e. *qPH19-HA-1*, *qPH20-HA-1*, *qPH21-HA-1*, *qGSA20-HA-1*, *qGSA21-HA-1*, *qGH21-HA-1*, *qDPHT3-HA-1*, *qDPHT4-HA-1*, *qDPHT5-HA-1*, *qDPHT6-HA-1*, *qDPHT7-HA-1*, and *qDCIT7-HA-1*) (Fig. 3A and B). All 12 QTLs peaked at the same peak QTL marker (Tag17408), with LOD scores ranging from 4.24 to 9.75, explaining 8.62% to 23.03% of the phenotypic variation for PH, CI, GSA, and GH (Fig. 3A and B). Another stable QTL region was also localized at the top of LG 1–1 (8.2–10.2 cM), including eight QTLs (i.e. *qPH19-HA-1*, *qPH20-HA-1*, *qGSA21-HA-1*, *qGH21-HA-1*, *qDPHT5-HA-1*, *qDPHT6-HA-1*, *qDPHT7-HA-2*, *qDCIT7-HA-1*) (Fig. 3A and B). The peak marker for these QTLs, Tag16173, colocalized at 9.2 cM, with LOD scores ranging from 4.20 to 9.40, explaining up to 20.13% phenotypic variation for PH, CI, GSA, and GH (Fig. 3A-B). The third and fourth stable QTL regions were located on the upper part of LG 1–1 (15.31–17.31 cM) and the bottom of LG 1–2 (85.11–89.21 cM) (Fig. 3A and C). The former region contained two QTLs associated with GSA (i.e. *qGSA20-HA-2* and *qGSA21-HA-3*), with the peak marker Tag46414, while the latter region possessed three QTLs related to CI (i.e. *qCI19-HA-1*, *qCI20-HA-1,* and *qCI21-HA-1*), all sharing the same peak marker, Tag26605 (Fig. 3A–C). The four stable QTL regions together likely determine the prostrate growth habit. Furthermore, we conducted additional QTL mapping analyses on the four stable QTLs using the average trait values across the four years. The results showed that the peak marker Tag17408 exhibited LOD scores ranging from 2.94 to 8.95, explaining 5.15% to 17.83% of the phenotypic variation for PH, CI, GSA, and GH (Fig. S3A). Similarly, Tag16173 showed LOD scores ranging from 2.57 to 9.55, explaining 4.41% to 18.29% of the phenotypic variation in the same traits (Fig. S3A). Tag46414 had an LOD score of 6.24 and explained 12.44% of the phenotypic variation for GSA (Fig. S3A). No significant QTLs were detected for Tag26605 (Fig. S3B).

To further evaluate the accuracy and reliability of the peak markers of four stable QTLs, KASP markers were developed and tested in F_1_, F_2,_ and BC_1_ populations (*n* = 366). Among the four KASP markers, only KASP_16173, corresponding to Tag16173, was successfully genotyped. In the validation populations, 83 individuals were heterozygous ‘A:T’ (representing *C. yantaiense*), while the remaining 269 were homozygous ‘T:T’ (representing *C. indicum*). Further analysis revealed that the KASP_16173 marker was tightly coupled with phenotypes of the validation population collected in 2021 and 2022, confirming the accuracy of QTL mapping. For instance, the PH of heterozygous individuals was 32.51% and 21.83% lower than that of homozygous ones (Fig. 3D). Similarly, the CI of heterozygous individuals was 37.35% and 25.65% lower than homozygous counterparts (Fig. 3D). A significant difference in GSA was also observed between the A:T and T:T genotypes, with the mean of GSA _A:T_ being 19.68% and 23.75% larger than GSA _T:T_ (Fig. 3D). For GH, the levels of individuals with A:T genotype were lower than those with T:T genotype (Fig. 3D). Lastly, although CP _A:T_ was higher than CP _T:T_, no significant difference was observed between the two genotypes (Fig. 3D). This finding was consistent with our previous QTL analysis, where Tag16173 was not identified as the peak QTL marker for CP. These results suggested that the region around Tag16173 may play a key role in regulating prostrate growth habit. Moreover, the development of this locus into the KASP marker will serve as a powerful tool for breeding new varieties with prostrate growth habit.

### Candidate genes of prostrate growth habit

**Figure 4:**
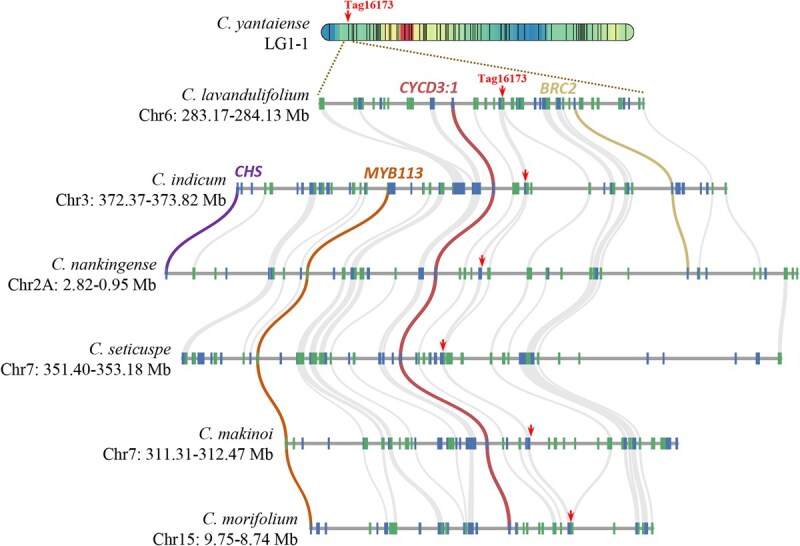
Corresponding regions of Tag16173 on the genomes of six *Chrysanthemum* species. The arrow indicates the position of the peak marker Tag16173 on the maternal genetic linkage map and the genomes of six *Chrysanthemum* species.

To identify candidate genes associated with prostrate growth habit, we aligned the biparental genetic maps to the genomes of two relatives, i.e. *C. morifolium* (hexaploid) and *C. lavandulifolium* (diploid) (Fig. S4) [9, 18]. Synteny analyses revealed that over 90% of the molecular markers could be successfully mapped to the genome maps of *C. morifolium* and *C. lavandulifolium*, demonstrating a high degree of conservation among the genomes of chrysanthemum species (Fig. S4). Therefore, the corresponding regions of Tag16173 on the genomes of six chrysanthemum species (i.e. *C. morifolium*, *C. lavandulifolium*, *C. seticuspe*, *C. nankingense*, *C. makinoi*, and *C. indicum*) were retrieved to identify candidate genes [9, 18–22]. As a result, 1.01 Mb, 0.96 Mb, 1.78 Mb, 1.87 Mb, 1.16 Mb, and 1.45 Mb regions on Chr 15 of *C. morifolium*, Chr 6 of *C. lavandulifolium*, Chr 7 of *C. seticuspe*, Chr 2A of *C. nankingense*, Chr 7 of *C. makinoi*, and Chr 3 of *C. indicum* were focused (Fig. 4). In this region, a total of 44 genes were detected across the genomes of six chrysanthemum species (Table S6). Among them, the best known were the homologs of the genes that regulate branching (e.g. *BRC2*) [23], control the balance between cell division and cell expansion and mediate the response of meristematic cells to cytokinin (e.g. *CYCD3;1*) [24], and are involved in anthocyanin biosynthesis (e.g. *MYB113* and *CHS*) (Fig. 4) [25, 26]. Genes located in this region all were potential candidate responsible for the formation of prostrate growth habit.

### Importance of *CyCYCD3;1* in the formation of prostrate growth habit

**Figure 5:**
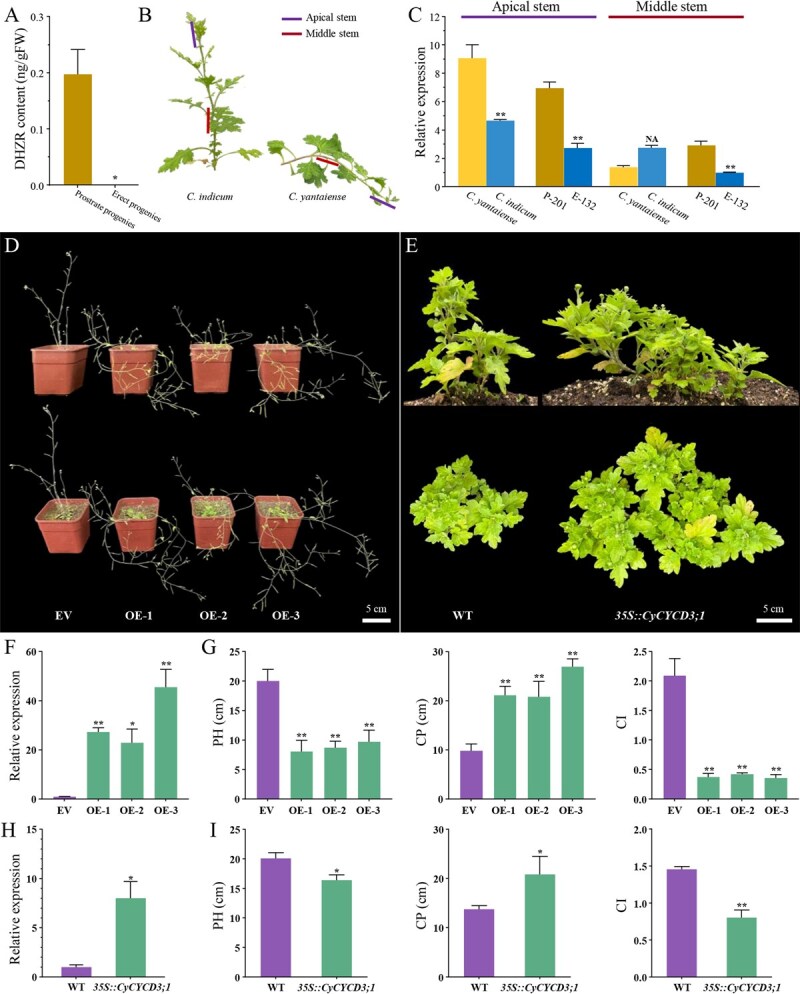
Expression and function of *CyCYCD3;1*. A DHZR content in prostrate and erect progenies. B Schematic diagram of sampling for qRT-PCR. C Relative expression of *CYCD3;1* in different stem parts of parents and progenies. D Phenotypes of *A. thaliana* overexpressing *CyCYCD3;1* and plant with EV. E Phenotypes of *C. morifolium* cv. ‘Pudidanfen’ overexpressing *CyCYCD3;1* and WT plant. F Relative expression of *CyCYCD3;1* in *Arabidopsis* overexpressing and plant with EV. G PH, CP, and CI of *CyCYCD3;1*-overexpressed transgenic Arabidopsis and plant with EV. H Relative expression of *CyCYCD3;1* in overexpressing plants and WT in *C. morifolium* cv. ‘Pudidanfen’*.* I PH, CP, and CI of *CyCYCD3;1*-overexpressed transgenic *C. morifolium* cv. ‘Pudidanfen’ and WT plant. Bar = 5 cm. Data are presented as means ± standard error from three independent replications. Statistical significance was determined by Students *t*-test: ^*^P < 0.05; ^**^P < 0.01.

Of these candidate genes, *CYCD3;1* attracted our special attention for three reasons. First, *CYCD3;1* and the peak marker Tag16173 were consistently linked within a region of approximately 112–160 kb across six chrysanthemum species (Table S7). According to the average recombination rate (the ratio of genetic map length to genome size) in chrysanthemum [9, 17, 27], this physical distance corresponds to a genetic distance of merely 0.05–0.07 cM, suggesting a tight linkage. Additionally, an amino acid insertion was found in the coding region of *CyCYCD3;1*, but was absent in its homologs in *C. indicum* (tetraploid) and the other six chrysanthemum species (Fig. S5). Second, the expression of *CYCD3;1* is regulated by cytokinins, and the content of Dihydrozeatin Riboside (DHZR), a highly active cytokinin, was significantly higher in prostrate progenies compared to erect progenies (Fig. 5A). Third, the homologs of *CyCYCD3;1* have been reported to promote cell proliferation, and have been proposed as a candidate gene regulating plant architecture [28, 29]. To gain some insights into its function, we first performed qRT-PCR analyses on both parents and progenies exhibiting the most extreme phenotypes (Fig. 5B and C). We found that the expression of *CyCYCD3;1* was 1.9-fold higher in the apical stem of *C. yantaiense* than in that of *C. indicum* (Fig. 5C). Similarly, its expression in the apical and middle stems of P-201 were 2.5-fold and 2.9-fold higher, respectively, than in those of E-132 (Fig. 5C). Clearly, the expression divergence of *CYCD3;1* strongly coincides with the formation of prostrate growth habit.

To investigate the function of *CyCYCD3;1*, we generated transgenic lines of *35S::CyCYCD3;1* in both *Arabidopsis thaliana* and *C. morifolium* cv. ‘Pudidanfen’(Fig. 5D and E). In T3 transgenic lines of *A. thaliana* (OE lines), the expression of *CyCYCD3;1* was significantly higher than in plants carrying the empty vector (EV) lines, confirming successful overexpression (Fig. 5F). Compared with EV lines, overexpression of *CyCYCD3;1* led to a 55.83% reduction in PH, a 133.16% increase in CP, and an 81.63% decrease in CI (Fig. 5G). Similarly, in *C. morifolium* cv. ‘Pudidanfen’, the expression level of overexpressed *CyCYCD3;1* was significantly higher than that in wild-type plants (WT) (Fig. 5H). Overexpression reduced PH by 18.41% and CI by 44.82%, while CP increased by 51.39% compared to WT lines (Fig. 5I). These findings indicated that *CyCYCD3;1* plays a critical role in regulating prostrate growth habit of *C. yantaiense*.

## Discussion

In this study, by conducting comprehensive genetic, morphological, physiological, and functional studies, we generated a hybrid population by crossing the prostrate-type *C. yantaiense* with the erect-type *C. indicum*, constructed the first high-density genetic linkage map for tetraploid chrysanthemum, detected four stable QTLs associated with prostrate growth habit-related traits, and revealed the mechanisms underlying prostrate growth habit in *C. yantaiense*. We observed that *C. yantaiense* and prostrate progenies exhibited higher CP, GSA, and stem pith cell number, but lower PH, CI, GH, and stem pith cell area compared to *C. indicum* and erect progenies. We pinpointed maternal LG1 as a key candidate region responsible for prostrate growth habit, with a stable QTL near the molecular marker Tag16173 confirmed by KASP and 44 candidate genes identified. In particular, we verified that one of the candidate genes, *CyCYCD3;1*, appears to be indispensable for the formation of prostrate growth habit. Our results not only enhance our understanding of the molecular mechanisms underlying prostrate growth habit, but also provide two valuable genetic maps for future molecular breeding in chrysanthemum. In particular, the KASP marker (KASP_16173) can be applied to marker-assisted selection (MAS) to improve the breeding efficiency of ground-cover chrysanthemum.

### An effective approach for exploring complex traits in polyploids

As a wild germplasm, *C. yantaiense* possesses valuable agronomic and ornamental characteristics, including trampling tolerance and strong ground-covering ability, making it an important genetic resource for breeding cultivated chrysanthemum [10]. However, its tetraploid nature and wild characteristics pose challenges for traditional hybrid breeding, such as prolonged breeding cycles and the introduction of undesirable traits along with target traits, leading to a decrease in ornamental quality [11]. Molecular marker-assisted breeding offers an effective means to overcome these breeding challenges. It is widely accepted that a high-density genetic linkage map serves as a fundamental resource for understanding the order of markers in polyploid species, especially those with unsequenced genomes. Combined with QTL analysis, molecular makers can be used to assist breeding and accelerate the utilization of polyploids. In hexaploidy chrysanthemum, this approach has been successfully used to identify QTLs associated with traits such as flower color, flowering time, flower diameter, ray floret length, ray floret number, corolla tube merged degree, and waterlogging tolerance [13–17]. Therefore, this method is also applicable to *C. yantaiense* for identification of genetic loci associated with the prostrate growth habit and for marker-assisted breeding.

Notably, quantitative traits such as prostrate growth habit are highly flexible and influenced by environmental factors, and thus conventional mapping methods that rely on one phenotypic data from single time point prone to high false-positive rates. Joint QTL mapping for multiple related traits can improve the efficiency and accuracy of QTL detection while enabling the differentiation between pleiotropy and tight linkage [30]. In this study, we first employed Super-GBS to construct a GBS reference genome, which provided more comprehensive SNP genotyping information and more accurate determination of heterozygous genotypes [31]. Furthermore, we not only focused on five traits related to prostrate growth habit over four years but also recorded dynamic phenotypic data at eight developmental stages. By integrating these data, we successfully identified four stable QTLs. Thereafter, we further developed KASP markers to evaluate the four stable QTLs, significantly enhancing the accuracy and reliability of our results. Given the robustness of our approach, it is highly likely that this strategy is reasonable and can be well applied to other polyploid species.

### Mechanisms underlying prostrate growth habit formation

Prostrate growth habit, an important agronomic trait with significant economic and ornamental value, has attracted great attention of breeders. It has been suggested that shoot gravitropism plays a key role in determining plant architecture by regulating tiller angle [1]. Several phytohormones, particularly auxin, and cytokinin, are believed to be central players in gravitropic signaling [32, 33]. Based mainly on studies in wild rice, many authors believed that *LA1* acted as a core regulator of tiller angle by influencing auxin transport during gravistimulation; in the *la1* mutant, the symmetric distribution of auxin in the tiller base was disturbed, leading to a large tiller angle [1]. Additionally, *PROSTRATE GROWTH 1* (*PROG1*) antagonistically interacted with *LA1* to modulate the tiller angle, thereby shaping the plant architecture [34]. Some other authors, however, insisted that the prostrate growth habit may be more closely related to cytokinin. For instance, Wang *et al.* found that in the *gi* mutant of rice, both the level of endogenous cytokinin as well as the size of panicle architecture were increased. Moreover, genes involved in the cytokinin-activated signaling pathway were enriched in the QTLs associated with peanut prostrate growth habit-related traits [8].

While these two scenarios are not mutually exclusive, our results supported the role of cytokinin in the formation of prostrate growth habit in *C. yantaiense*. On one hand, a significant difference in cytokinin content was detected between the stems of prostrate and erect plants. On the other hand, *CYCD3;1*, a gene responsive to cytokinin, was identified within the QTL associated with prostrate growth habit, and its function was confirmed by transgenic experiments. The importance of auxins, however, cannot be supported or rejected in this study. Therefore, functional studies of other genes within the identified QTLs are urgently needed in future research.

## Materials and methods

### Plant materials and hybridization

*C. yantaiense* and *C. indicum* were collected from the coastal area of Shandong Province and the Shennongjia National Nature Reserve in Hubei Province, China, respectively. We performed crosses between *C. yantaiense* (maternal plant) and *C. indicum* (paternal plant) over two consecutive years, resulting in 199 F_1_ progenies in 2018 and 143 in 2019. The hybrid seeds were grown in the chrysanthemum nursery at the National Engineering Research Center for Floriculture (Beijing, China), which provided suitable climatic conditions, including appropriate temperature, photoperiod, and soil moisture. Based on phenotypic observations in 2019, plants with intermediate plant architecture (i.e. F_1_–3 and F_1_–45) in the F_1_ generation were selected to produce the F_2_ population, and F_1_–3 was chosen for backcrossing with the maternal plant to generate the BC_1_ population. Methods for cultivating the F_2_ and BC_1_ generations followed the same procedures used for the F_1_ generation. Cultivation and management practices adhered to conventional field cultivation methods.

### Phenotypic data analysis

Phenotypic data of 526 individuals, including parents, 342 F_1_ individuals, 148 F_2_ individuals, and 34 BC_1_ individuals, were collected in the field. Four years (from 2019 to 2022) of phenotypic evaluation were conducted for two parents and the F_1_ mapping population (199 individuals), and two years (2021 and 2020) of phenotypic evaluation were performed for the validation population (including 182 F_1_ individuals, 148 F_2_ individuals, and 34 BC_1_ individuals). The five traits related to prostrate habit including plant height (PH), crown width of the plant (CP), creeping index (CI), gravitropic set-point angle of branching (GSA), and growth habit (GH) were evaluated. PH was measured as the vertical height from the base to the tallest point of the plant, excluding abnormal branches. CP was defined as the average distances in the east–west and north–south directions of the plant. CI represents the ratio of PH to CP. GSA is the angle between the secondary branches and the direction of negative gravitropism. GH indicates the degree of prostrate growth habit, as detailed in Table S8. PH, GH, and CI were evaluated once per individual plant, while CP and GSA were assessed with two and three technical replicates, respectively. GSA was measured repeatedly over three years, and other traits were evaluated over four years. In 2021, PH, CP, and CI were also measured for the F_1_ mapping population at eight developmental stages (i.e. 45, 55, 65, 75, 85, 95, 105, and 115 days after transplanting). Heritability analysis was performed using SEA software [35], and statistical analyses were conducted with the R packages ‘psych’, and ‘corrplot’.

### Histological observation

The apical stems (from the first to third internodes) of parents and progenies with extreme phenotype (P-201 and E-132) were collected during the vegetative growth stage and immediately fixed for 24 h at 4°C in FAA fixative (Solarbio G2350), each with three biological replicates. Paraffin sections were prepared following the method reported by Li *et al.* [36]. Tissue sections, cut to a thickness of 10–12 μm (Leica RM 2016, Wetzlar, Germany), were stained with safranin O-fast green. The samples were then observed using an optical microscope (Soptop CX40P, Sungrant, Suzhou, China). Cell number and cell area were measured using ImageJ (1.53a) software [37].

### DNA extraction and sequencing

Healthy leaves were collected from parents and 199 individuals of the F_1_ mapping population obtained in 2018, rapidly frozen in liquid nitrogen and stored at −80°C. DNA was extracted and measured using a Plant Genomic DNA Extraction Kit from TIANGEN (Beijing, China) and Nanodrop (Thermo Scientific), respectively. The DNA samples were further diluted to 50 ng/μl. DNA was digested with the methylation-sensitive restriction enzymes PstI, and MspI, followed by the construction of barcoded libraries for each sample. Library concentrations were quantified using the dsDNA High Sensitivity Assay Kit on a Qubit 2.0 fluorometer. Fragments smaller than 300 bp were removed from the pooled libraries using Sera-Mag SpeedBeads (GE Healthcare Life Sciences) and a magnetic stand [38]. High-quality samples were sequenced on Illumina NovaSeq platform (paired-end 150 bp).

### Genotyping and genetic map construction

The raw data were first split based on barcodes and then trimmed to remove adapters, barcodes, and low-quality bases. The clean reads were aligned to the GBS reference generated using the UGbS-Flex pipeline with Bowtie2 [38], and SNPs and INDELs were called using GATK version 3.4 with default parameters [39]. In this study, the ‘double pseudo-testcross’ mapping strategy was adopted to construct linkage maps in two tetraploid F_1_ population. Specifically, the tetraploids were regarded as diploids, and only markers fitting the testcross configuration (i.e. one parent homozygous and the other heterozygous at a given locus) and exhibiting a 1:1 segregation ratio in the progeny were retained for genetic map construction. Firstly, SNPs with allelic depth (AD) < 8 were marked as missing (−), and we also removed adjacent SNPs. Secondly, SNPs with three or more alleles, or with allele frequencies below 0.1 or above 0.9 were excluded. SNPs with an AD_ref(reference allele)_/AD_alt(alternate allele)_ ratio ≥ 10 were scored as A (homozygous for the parent allele), AD_ref_/AD_alt_ ≤ 0.10 as B (homozygous), 10 > AD_ref_/AD_alt_ > 4 as D (ambiguous A or H) and 0.25 > AD_ref_/AD_alt_ > 0.1 as C (ambiguous B or H). Loci with other ratios were scored as H (heterozygous). Thirdly, Chi-square tests were performed to assess each marker's deviation from expected Mendelian segregation ratios, and markers with significant distortion (*P*-value ≤1e^−10^) were excluded. The remaining SNPs were grouped by their segregation ratio and parental genotype. Since the focus was on constructing separate parental linkage maps rather than a consensus map of both parents, heterozygous markers in both parents were removed. SNPs with genotypic score A or B in parent 1 and H in parent 2, segregating as A:H or B:H (1:1) marker were combined, and B scores were converted to A scores to create the paternal (AH) dataset (Table S3). Similarly, H:A and H:B (1:1) markers were combined, and B scores were converted to A scores to form the maternal (HA) dataset (Table S4). The filtering criteria for INDELs were the same as those for SNPs. The maternal and paternal genetic maps were constructed using a combination of MSTMap [40] and MAPMAKER [41] modified as described by Qi *et al.* [38]. Both datasets were set as ‘backcross’ population types. Cosegregating markers were added to the framework map at the position of their representative marker with an in-house-developed Python script. The maps were visualized with MapChart [42].

### QTL analysis for prostrate growth habit-related traits

QTLs for prostrate growth habit-related traits were identified using the parental linkage maps derived from the *C. yantaiense* × *C. indicum* F_1_ population. To evaluate the stability of marker-trait associations, QTL mapping was performed using Windows QTL Cartographer version 2.5 [43], with the composite interval mapping model (CIM) and phenotypic data collected over four years. In both maternal and paternal maps, the population type was set to 'B2' for QTL analysis, with a walking speed of 0.5 cM used for QTL identification. The LOD threshold for significant QTL (*P* ≤ 0.05) was determined based on 1000 permutations. QTLs were designated with the prefix 'q', followed by the trait abbreviation, genetic map code (HA/AH), and the ordinal number of the associated QTL. Dynamic QTLs were labeled by adding ‘D’ before the trait abbreviation. Full names were presented in italics.

### Development and analysis of KASP markers

The GBS tags containing SNPs associated with stable QTLs for prostrate growth habit-related traits were selected, and primers were designed for KASP genotyping. A total of 366 individuals, including parents, F_1_, F_2_, and BC_1_ populations, were randomly selected for KASP genotyping. Primer sets were designed and synthesized by LGC Genomics (Hoddesdon, UK) (Table S9). Genotyping was performed through a two-step PCR amplification of the target region under the following conditions: initial activation at 94°C for 15 min; amplification with denaturation at 94°C for 20 s, followed by annealing and extension at 61°C–55°C gradient for 60 s (with a 0.6°C decrease in each cycle) for 10 cycles; and then, amplification of fluorescence signals with denaturation at 94°C for 20 s, followed by annealing and extension at 55°C for 60 s for 26 cycles. Fluorescence signals were converted into analyzable data using a FLUOstar® Omega Microplate Reader (BMG Labtech, Germany), and genotyped using Kraken™ (LGC) soft (www.lgcgroup.com/software). The genotyping data were visualized using SNPviewer (LGC Genomics).

### Measurement of phytohormones

The apical stems from three extreme prostrate progenies (P-201, P-177, and P-84), and three extreme erect progenies (E-132, E-149, and E-15) were selected from the F_1_ population for phytohormones quantitation. The samples were collected at the vegetative stage, 60 days after transplanting, and immediately frozen in liquid nitrogen and stored at −80°C. LC–MS/MS analysis was conducted using an ultra-performance liquid chromatography-electrospray ionization tandem mass spectrometry (UPLC-ESI-MS/MS) system (ExionLC™ AD, Sciex, Washington, DC, USA; QTRAP®6500+; Applied Biosystems; Redwood CA, USA) [44–46]. Phytohormone quantification was performed by Metware (Wuhan Metware Biotechnology Co., Ltd., Wuhan, China) (http://www.metware.cn/). Detailed methodologies were described in a previous study [47].

### Synteny analysis and candidate gene prediction

Due to the absence of reference genome for *C. yantaiense*, the SNP markers in biparental linkage maps were anchored to the genome maps of *C. morifolium* and *C. lavandulifolium* [9, 18], following methods described by Song *et al.* [17]. The results were visualized using the Circos tool (http://circos.ca) [48]. Furthermore, the corresponding regions of Tag16173 on the genomes of six chrysanthemum species (i.e. *C. morifolium*, *C. lavandulifolium*, *C. seticuspe*, *C. nankingense*, *C. makinoi*, and *C. indicum*) were retrieved to identify candidate genes [9, 18–22, 49]. The functions of the candidate genes were predicted by performing BLAST searches against *A. thaliana*.

### Gene cloning and expression analysis

Total RNA was isolated from the apical and middle stems of parents and progenies with extreme phenotypes (i.e. P-201 and E-132) at the vegetative growth stage (60 days after transplanting) using a Total RNA extraction kit (Omega, GA, USA) and PrimeScript™ RT Reagent Kit with gDNA Eraser (Perfect Real Time) (TaKaRa, Shiga, Japan), each with three biological replicates. The generated cDNA was stored at −20°C for subsequent gene cloning and quantitative real-time PCR (qRT-PCR).

The CDS of *CyCYCD3;1* gene was amplified using gene-specific primers (Table S10). qRT-PCR was performed using the PikoReal real-time PCR system (Thermo Fisher Scientific, MA, USA). The qRT-PCR procedure and internal reference gene (*CmSAND*) were previously described [50]. Relative expression levels were calculated using the 2^-ΔΔCt^ method [51].

### Generation of transgenic Arabidopsis and Chrysanthemum

The CDS of *CyCYCD3;1* was inserted into the plant expression vector pSuper1300, which contains kanamycin and hygromycin resistance genes. The recombinant vectors were then transformed into *Agrobacterium tumefaciens* (GV3101) [52]. Arabidopsis transformation was performed using the floral dip method (Clough and Bent, 1998). PCR amplification was conducted using primers listed in Table S10 to identify the transgenic plants. Positive lines were selected with hygromycin B (50 mg/L). Arabidopsis plants transformed with the EV served as the negative control. For *C. morifolium* cv. ‘Pudidanfen’, transgenic plants were generated via the leaf disc transformation. The transformation was induced using MS medium supplemented with 1 mg/L 6-BA, 0.7 mg/L NAA, and 400 mg/L Carb, and selected on MS medium containing 5 mg/L Hyg B and 400 mg/L Carb. PCR was performed to identify positive plants using primers from Table S10. The expression level of *CyCYCD3;1* was measured by qRT-PCR, each with three biological replicates.

## Supplementary Material

Web_Material_uhaf129

## Data Availability

All the data can be found in the main text or the supplements. The raw sequencing data of the population reported in this paper have been deposited in the NCBI BioProject under accession number PRJNA1214410.
