# Supplementary material for: QTL detection and candidate gene identification for prostrate growth habit in interspecific crosses of wild chrysanthemum (Chrysanthemum yantaiense × C. indicum)
Source: Hortic Res. 2025 May 21;12(8):uhaf129. doi: 10.1093/hr/uhaf129 (PMC12265470; doi:10.1093/hr/uhaf129)
Supplement: Web_Material_uhaf129 [file web_material_uhaf129.zip › Supplementary Figures.docx]

| 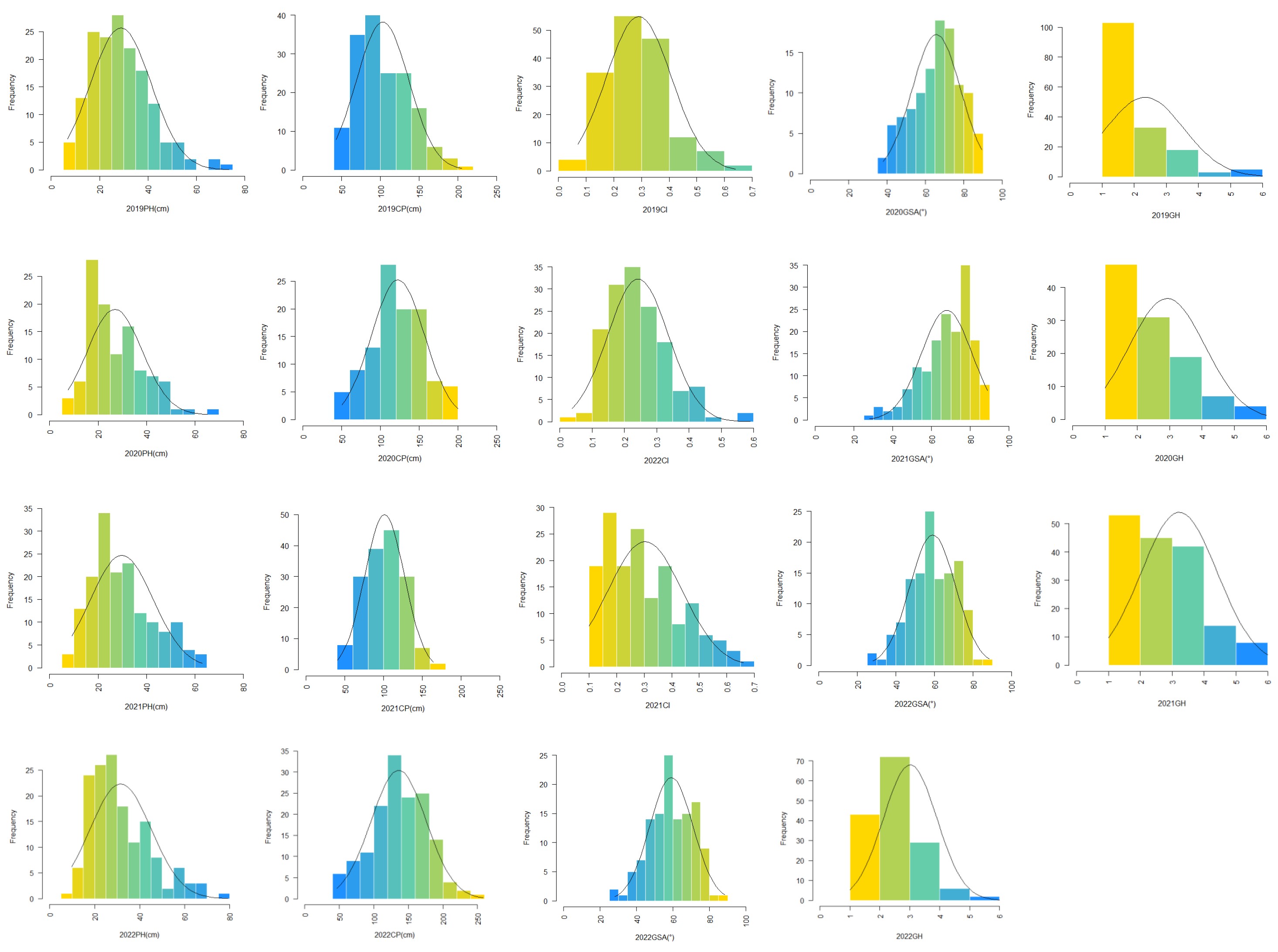 |
| --- |
| Fig. S1 Distribution of PH, CP, CI, GSA, and GH in the F_1_ mapping population from 2019 to 2022. PH, Plant height; CP, Crown width of the plant; CI, creeping index; GSA, gravitropic set-point angle of branching; GH, growth habit. |

| 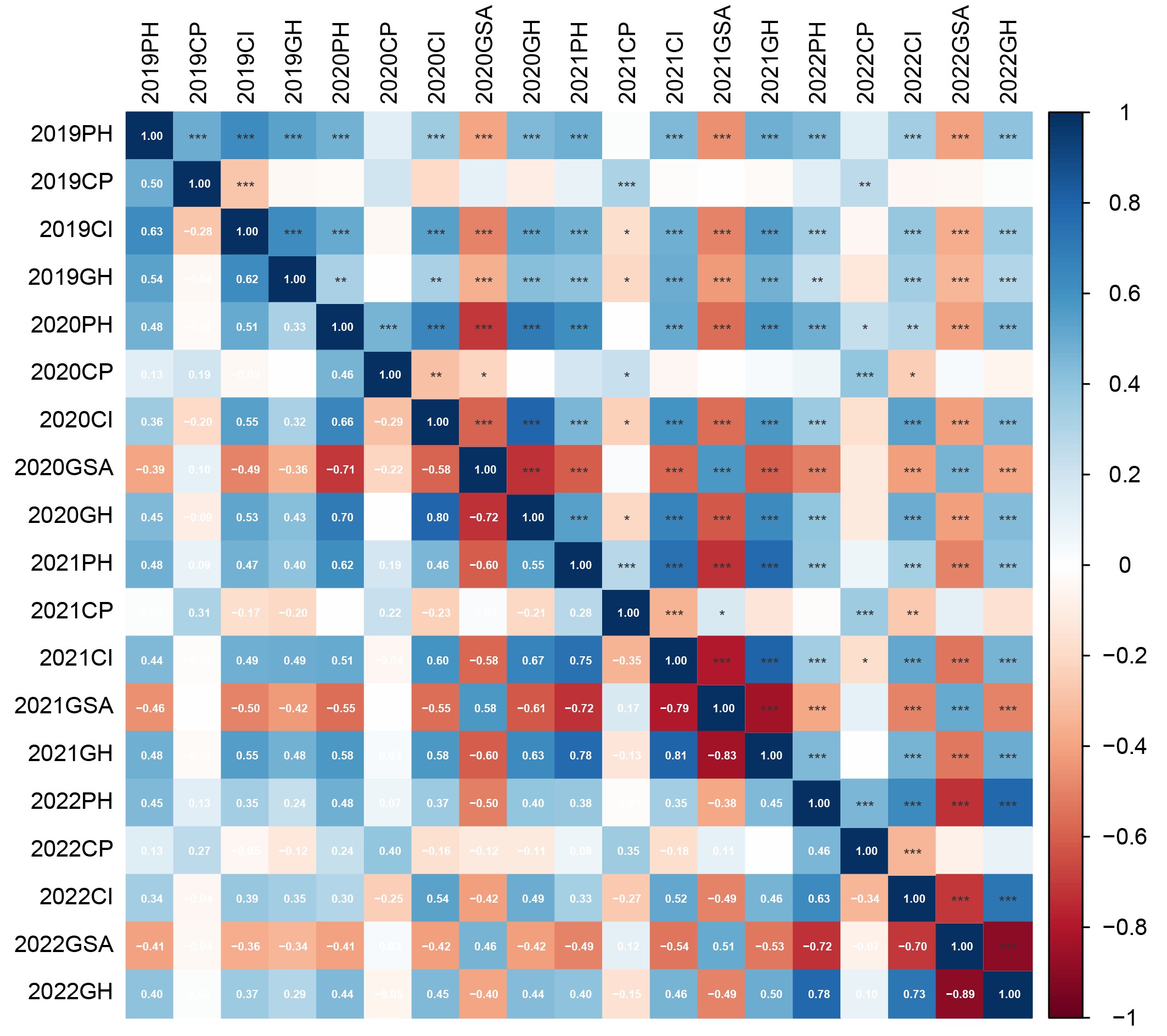 |
| --- |
| Fig. S2 Pearson correlation coefficients of prostrate growth habit-related traits from 2019 to 2022. P values of correlation analysis: ^*^*P* < 0.05; ^**^*P* < 0.01; ^∗∗∗^*P* < 0.001; ^∗∗∗∗^*P* < 0.0001. PH, Plant height; CP, Crown width; CI, creeping index; GSA, gravitropic set-point angle of branching; GH, growth habit. |

| 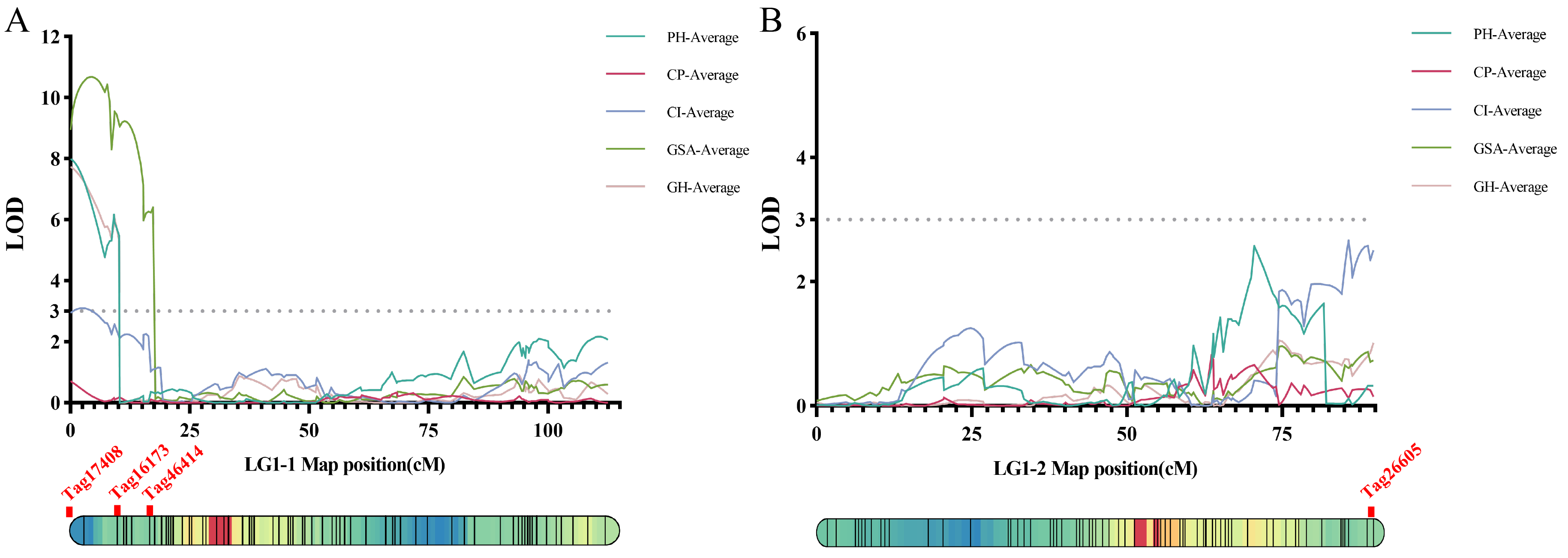 |
| --- |
| **Fig. S3** Co-localized QTLs of prostrate growth habit-related traits using four-year average trait values. A Co-localized QTLs of prostrate growth habit-related traits in LG1-1. B Co-localized QTLs of prostrate growth habit-related traits in LG1-2. |

| 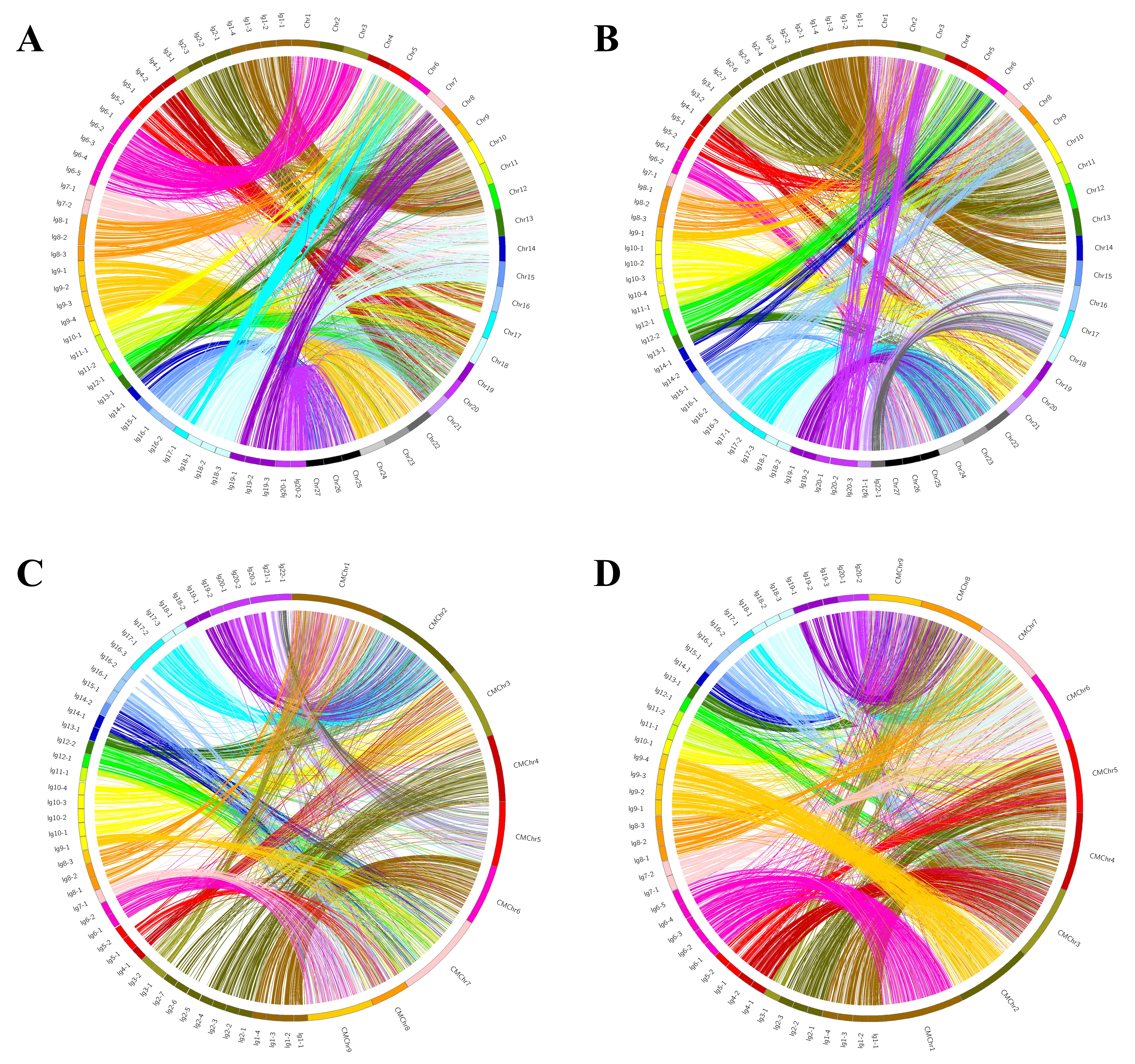 |
| --- |
| Fig. S4 Syntenic relationships between biparental genetic maps and the genomes of *C. morifolium* and *C. lavandulifolium*. A Synteny between the maternal linkage map(HA map) and physical map of *C.morifolium*. B Synteny between the paternal linkage map(AH map) and physical map of *C.morifolium*. C Synteny between the maternal linkage map(HA map) and physical map of *C.* *lavandulifolium*. D Synteny between the paternal linkage map(AH map) and physical map of *C.* *lavandulifolium*. |
|  |
| 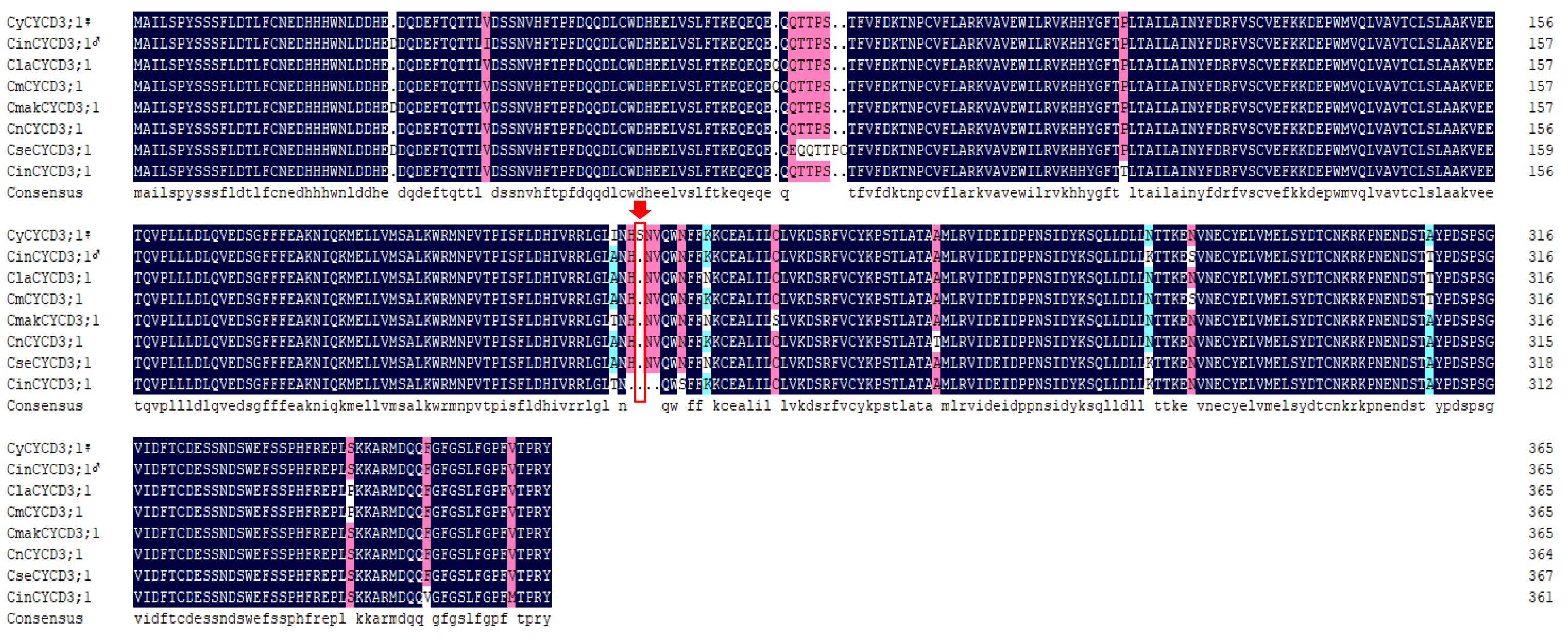 |
| Fig. S5 Multiple sequence alignment of the amino acid sequences of *CYCD3;1* in eight *Chrysanthemum* species. The red arrow highlights the unique amino acid obtained in *CyCYCD3;1*. |
